# Mechanical thrombectomy for post-thrombotic venous obstruction is associated with venous wound healing

**DOI:** 10.1016/j.jvsv.2026.102625

**Published:** 2026-09-19

**Authors:** Nicolas J. Mouawad, Steven Abramowitz, Atif Baqai, Angelo Marino

**Affiliations:** a Division of Vascular and Endovascular Surgery, Department of Surgery, McLaren Health System, Bay City, MI; b MedStar Health, Washington, DC; c Ascension Illinois Heart and Vascular Institute, Chicago, IL; d Yale University School of Medicine, New Haven, CT

**Keywords:** Chronic venous wounds, Mechanical thrombectomy, Deep vein thrombosis, Venous obstruction, Post-thrombotic syndrome

## Abstract

**Objective:**

To evaluate the association between mechanical thrombectomy (MT)—combined with standard wound care and adjunctive endovenous interventions, as indicated—and venous wound healing in patients with post-thrombotic venous obstruction.

**Methods:**

This multicenter, retrospective analysis included adults with venous wounds secondary to lower-extremity deep vein thrombosis. MT was performed with one or more of the following devices (Stryker): ClotTriever System, ClotTriever BOLD catheter, or VenaCore catheter in native vessels, or RevCore catheter (Stryker) within occluded stents. Adjunctive venoplasty and stenting were performed as indicated. Standard wound care continued until wound healing or latest follow-up. Technical success was defined as crossing the lesion and deploying the study device; clinical success as any decrease from baseline in wound area.

**Results:**

Twenty-two patients with 37 wounds on 29 limbs were treated between August 2021 and September 2024. Mean age was 59.0 ± 12.8 years, and 72.7% were male. More than half had failed conservative wound care before referral; three had been referred for amputation. Median wound duration was 13.0 [interquartile range (IQR), 6.3-45.0] months. Technical success was achieved in 100% of cases; all procedures were single session, with 91.7% performed outpatient. Stenting was performed in 15 of 29 limbs (51.7%) and venoplasty in 23 of 29 (79.3%). Among the 14 limbs without new stenting at the index procedure, two underwent MT alone and 11 received adjunctive venoplasty only. No adjunctive thrombolytics were used. Hospital stay (median, 2; IQR, 2-2.75 days) was required following six procedures. At latest follow-up (median, 2.8; IQR, 2.0-3.5 months), all wounds were healed or healing, meeting the clinical success definition. Among the 25 wounds with available area data, mean wound area decreased from 22.0 ± 30.9 cm^2^ at baseline to 5.1 ± 20.8 cm^2^, with a mean per-wound area reduction of 91.4%; 76% (19/25) fully healed, and the remaining wounds showed a mean per-wound area reduction of 65.6%.

**Conclusions:**

In this multicenter retrospective analysis, all patients with post-thrombotic venous wounds were healed or healing at latest follow-up after MT, with a 91% mean per-wound area reduction. These findings suggest that MT—combined with standard wound care and adjunctive endovenous interventions as indicated—may play an important role in treating recalcitrant post-thrombotic venous wounds.

**Article Highlights**

• **Type of Research:** Multicenter retrospective cohort study
• **Key Findings:** Twenty-two patients with chronic, refractory, deep vein thrombosis-related, venous wounds underwent mechanical thrombectomy with or without adjunctive endovenous interventions and without thrombolytics. Technical and clinical success were achieved in all cases. At 3-month follow-up, 76% of wounds were fully healed; mean wound area decreased from 22.0 to 5.1 cm^2^, with a mean per-wound area reduction of 91.4%.
• **Take Home Message:** These multicenter findings suggest that mechanical thrombectomy may be an important component of the treatment for chronic venous wounds secondary to post-thrombotic venous obstruction when combined with standard wound care and indicated adjunctive endovenous interventions.

Following a deep vein thrombosis (DVT), residual thrombus may evolve into post-thrombotic obstruction (PTO).^1^, ^2^, ^3^ Together with venous reflux and persistent vein wall inflammation, PTO may contribute to chronic venous hypertension, impaired venous outflow, venous congestion, and tissue hypoxia.^2^, ^3^, ^4^ These hemodynamic abnormalities underlie the development of post-thrombotic syndrome (PTS), a chronic form of venous disease that affects 20% to 50% of patients after proximal DVT.^1^^,^^5^^,^^6^ Although most patients with PTS experience symptoms such as pain, edema, and skin changes, approximately 5% to 10% develop severe PTS, which may manifest as recalcitrant venous wounds.^1^^,^^3^ Under the Clinical-Etiology-Anatomy-Pathophysiology (CEAP) classification system, these wounds are classified as class 6 (C6) disease (Fig 1, Baseline).^3^^,^^7^^,^^8^

**Fig 1:**
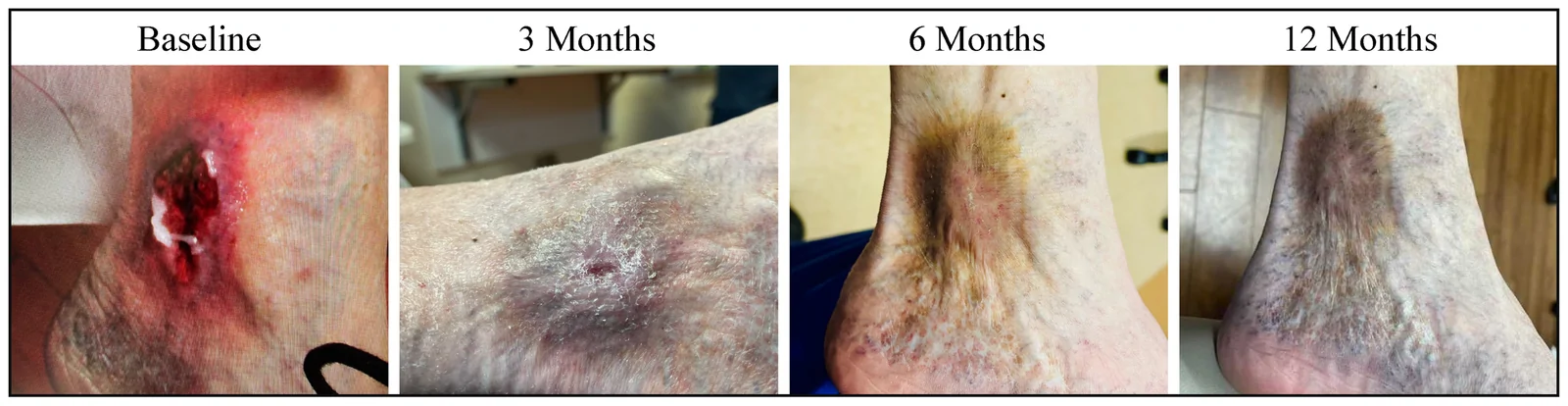
A 64-year-old female with a history of bilateral leg deep vein thrombosis (DVT), caval thrombus, inferior vena cava (IVC) filter placement, chronic obstructive pulmonary disease (COPD), malnutrition, and a mood disorder presented with bilateral leg swelling and bilateral venous ulcers. She underwent bilateral iliofemoral ClotTriever-based mechanical thrombectomy (MT) with FlowTriever use at the IVC filter site, aggressive serial common iliac vein ballooning, and bilateral venoplasty without stenting. Standard wound care continued postprocedure, and both venous wounds healed at latest follow-up. The left venous wound, pictured, was healing within 3 months and fully healed within a year.

Current international guidelines recommend a stepwise approach to managing advanced chronic venous disease (CEAP classes C4-C6), encompassing conservative care, superficial venous ablation, and endovascular reconstruction.^7^ Iliac vein and inferior vena cava stenting are recommended as first-line interventions for patients with underlying venous outflow obstruction,^7^ and iliac vein stenting specifically has been shown to accelerate venous wound healing.^9^ Despite this range of options, available therapies remain largely palliative, addressing the consequences of venous hypertension without removing the underlying PTO.^2^^,^^5^

Mechanical thrombectomy (MT) is a minimally invasive procedure used to remove thrombus and presents a novel therapeutic option for C6 disease.^10^ By extracting PTOs from the venous system, MT has the potential to improve venous outflow, reduce venous pressure, and ultimately promote resolution of C6 disease.^11^^,^^12^ Although MT is well established in the management of DVT,^10^^,^^13^ its application in C6 disease remains relatively unexplored. Previous retrospective analyses demonstrated that single-session MT can remove chronic PTOs without thrombolytics and achieve complete or near-complete venous wound healing, even in patients with long-standing disease who had been referred for amputation.^11^^,^^12^^,^^14^

In this study, we evaluate the association between MT—in combination with standard wound care and with or without adjunctive endovenous interventions—and wound healing outcomes in a multicenter cohort.

## Methods

This multicenter, retrospective analysis included patients who were ≥18 years of age, classified with C6 disease secondary to lower-extremity DVT, and treated with MT at one of four participating hospitals using one or more of the following devices (Stryker): ClotTriever System or ClotTriever BOLD in native vessels, VenaCore catheter for chronic or adherent obstructions, or RevCore catheter within occluded stents. This represents an all-comer series: all consecutive patients meeting the above criteria during the study period were included. No formal sample size justification was performed, as this was a descriptive retrospective analysis.

The following describes the treatment protocol at a representative center; participating centers followed similar protocols with minor procedural differences. Patients had been referred from a regional network of wound care centers after failure of compression therapy and local wound care. Following referral, patients were managed with continued leg elevation, compression therapy, local wound care, and close follow-up per standard of care. Preprocedural bilateral lower-extremity venous duplex ultrasound with reflux assessment was performed. When identified, refluxing superficial veins and pathologic perforating veins were treated before MT. Patients subsequently underwent MT with or without adjunctive venoplasty and/or stenting; both device selection and adjunctive procedures were determined by operator discretion based on vascular workup findings. Intravascular ultrasound (IVUS) was used in all procedures to assess luminal gain following thrombectomy.

Technical success was defined as the ability to cross the target lesion and deploy the study device. Clinical success was defined as any decrease from baseline in wound area at latest follow-up. Complete wound healing was defined as full epithelialization (wound area = 0 cm^2^); healing was defined as a decrease in wound area from baseline without full epithelialization; and failure was defined as stable or increasing wound area. Follow-up occurred at wound care clinic visits at intervals between 1 and 11 months post-MT. Additional data collected included demographics, comorbidities, and procedural characteristics. The presented data include measurements collected through the visit at which full wound healing was achieved or, if the wound persisted, the last available follow-up visit.

Descriptive statistics were used throughout. Means and standard deviations were generated for continuous normally distributed variables; medians and interquartile ranges (IQRs) were calculated for skewed variables. Counts and percentages were used for categorical variables. Comorbidities were extracted from documented medical history and are reported as raw counts only given incomplete historical documentation across the cohort. Data collection and analysis were performed using Excel (Microsoft). The conduct of this study was governed by the Declaration of Helsinki. Patient data were deidentified before analysis; therefore, Institutional Review Board approval was waived.

## Results

From August 2021 to September 2024, a total of 22 patients with CEAP class C6 disease underwent index MT procedures with at least one study device at one of four participating centers; bilateral disease was treated in some cases, yielding 29 treated limbs. Where available, patient-level characteristics are reported for the 22 unique patients and limb-level data for the 29 treated limbs. Baseline and procedural characteristics are summarized in Table I. Mean patient age was 59.0 ± 12.8 years, and 72.7% of patients were male. Mean body mass index was 34.5 ± 9.0 kg/m^2^. Twelve of 22 patients with available data were referred following failure of conservative wound care; three of these 12 patients had been referred for primary amputation before evaluation. The most commonly documented comorbidities were previous DVT (n = 16), PTS (n = 5), chronic obstructive pulmonary disease (n = 4), pulmonary embolism (n = 3), and hypertension (n = 3). Previous anticoagulation therapy was documented in eight of 13 patients with available data (61.5%). A pre-existing venous stent was documented in nine of 22 patients (40.9%).

**Table I:** Baseline and procedural characteristics

Patient characteristics	N = 22 (%)
Age, mean (SD), years	59.0 ± 12.8
Body mass index, mean (SD), kg/m^2^	34.5 ± 9.0
Male	16 (72.7)
Venous wound characteristics	N = 37
Duration, median [IQR], months	13.0 [6.3-45.0]
Baseline wound area, mean (SD), cm^2^	22.0 ± 30.9
Procedural characteristics	N = 29 (%)
Technical success^a^	29 (100)
Study device used^b^	
ClotTriever System	15 (51.7)
ClotTriever BOLD	4 (13.8)
RevCore	9 (31.0)
VenaCore	5 (17.2)
ProTrieve Sheath	7 (24.1)
Adjunctive procedures	
Thrombolytics	0
Stent placement	15 (51.7)
Venoplasty	23 (79.3)

*IQR*, Interquartile range; *SD*, standard deviation.

Data presented as n (%) or median [IQR] as appropriate.

a Technical success defined as the ability to cross a lesion and deploy the study device.

b More than one type of study device could be used per procedure.

A total of 37 wounds on 29 limbs were included. Median duration of venous symptoms was 13.0 (IQR, 6.3-45.0) months, and mean baseline wound area was 22.0 ± 30.9 cm^2^. Obstruction location data were available for 23 of 29 treated limbs; the most frequently involved segments were the left common iliac vein (34.8%), left external iliac vein (34.8%), and right common iliac vein (34.8%), followed by inferior vena cava (26.1%) and right external iliac vein (26.1%). Infrainguinal involvement was present in 52.2% of limbs with available data, reflecting the complex, multisegmented nature of post-thrombotic disease in this cohort.

Technical success, defined as the ability to cross the target lesion and deploy the study device, was achieved in 100% of cases. The ClotTriever System was used in 15 limbs (51.7%), ClotTriever BOLD in four (13.8%), RevCore in nine (31.0%), and VenaCore in five (17.2%); more than one device type was used in 10 (34.5%) limbs. No thrombolytics were administered in any case. The Protrieve sheath was used for embolic capture in seven (24.1%) limbs; embolic debris was captured in all cases with available data. General anesthesia was used in nine of 18 patients with available data (50.0%). Intraoperative estimated blood loss was recorded for five limbs across four patients, with a median of 30 (IQR, 20-40) mL lost. All procedures were completed in a single session, with 91.7% performed in an outpatient setting.

Adjunctive procedures often co-occurred within the same limb. At the limb level, stenting was performed in 15 of 29 limbs (51.7%) and venoplasty in 23 of 29 limbs (79.3%). At the patient level, nine of 22 patients (40.9%) had a documented pre-existing stent at baseline; among these, six (66.7%) received an additional stent during the index procedure or a subsequent reintervention. Of the 13 patients without a documented baseline stent, eight (61.5%) received their first venous stent during the index thrombectomy. Among the 14 limbs that did not receive new stenting at the index procedure, two underwent MT alone (no adjunctive procedure) and 11 underwent MT with adjunctive venoplasty; venoplasty status was undocumented for one additional limb.

Median thrombus removal was 90.0% (IQR, 87.5%-92.5%) based on intraoperative venography. Quantitative IVUS measurements of luminal gain were recorded only in the RevCore subgroup, in which inner lumen diameter increased by 69.8% ± 22.3% across 12 treated stents in nine limbs from seven patients. Across the broader cohort, operators reported IVUS-based luminal gain consistent with the RevCore findings, though without systematic documentation.

Hospitalization was required following six procedures, with a median stay of 2 (IQR, 2-2.75) days. No intensive care unit stays were required. No procedure-related complications or adverse events were identified, consistent with findings from the largest prospective registry of ClotTriever-based MT for DVT.^10^ Three postprocedural events were noted but classified as unrelated to the index procedure. One patient remained hospitalized following the procedure to receive intravenous antibiotics for a pre-existing infected venous wound. A second patient was readmitted on postoperative day 37 for worsening right lower-extremity swelling secondary to occlusion of the right iliac stent placed at the index procedure; this event was considered unrelated to the index procedure. A third patient experienced occlusion of the left iliac stent approximately 6 months following the index thrombectomy and underwent successful reintervention, similarly considered unrelated to the index procedure. One patient treated with RevCore had residual stent stenosis identified at the conclusion of the procedure, which was noted for surveillance and did not constitute a procedural complication.

Clinical success outcomes are summarized in Table II. Clinical success was achieved for all 37 wounds (100%). Of the 25 wounds with area measurements at latest follow-up (median, 2.8; IQR, 2.0-3.5 months), mean wound area decreased from 22.0 ± 30.9 cm^2^ at baseline to 5.1 ± 20.8 cm^2^ at latest follow-up, with a mean per-wound area reduction of 91.4% (Fig 2). Complete wound healing was achieved in 76% (19/25) of wounds, and the remaining six wounds showed a mean per-wound area reduction of 65.6%. Of the remaining wounds without area measurements, all were observed to be healing.

**Table II:** Clinical success and safety outcomes

Wound characteristics	N = 37 (%)
Clinical success	37 (100)
Complete wound healing	19/25 (76.0)
Follow-up wound area, mean (SD), cm^2^	5.1 ± 20.8
Per-wound area reduction, %	91.4
Safety outcomes	
Procedure-related complications	0
ICU stay required	0

*ICU*, Intensive care unit; *SD*, standard deviation.

Categorical data are shown as number (%).

**Fig 2:**
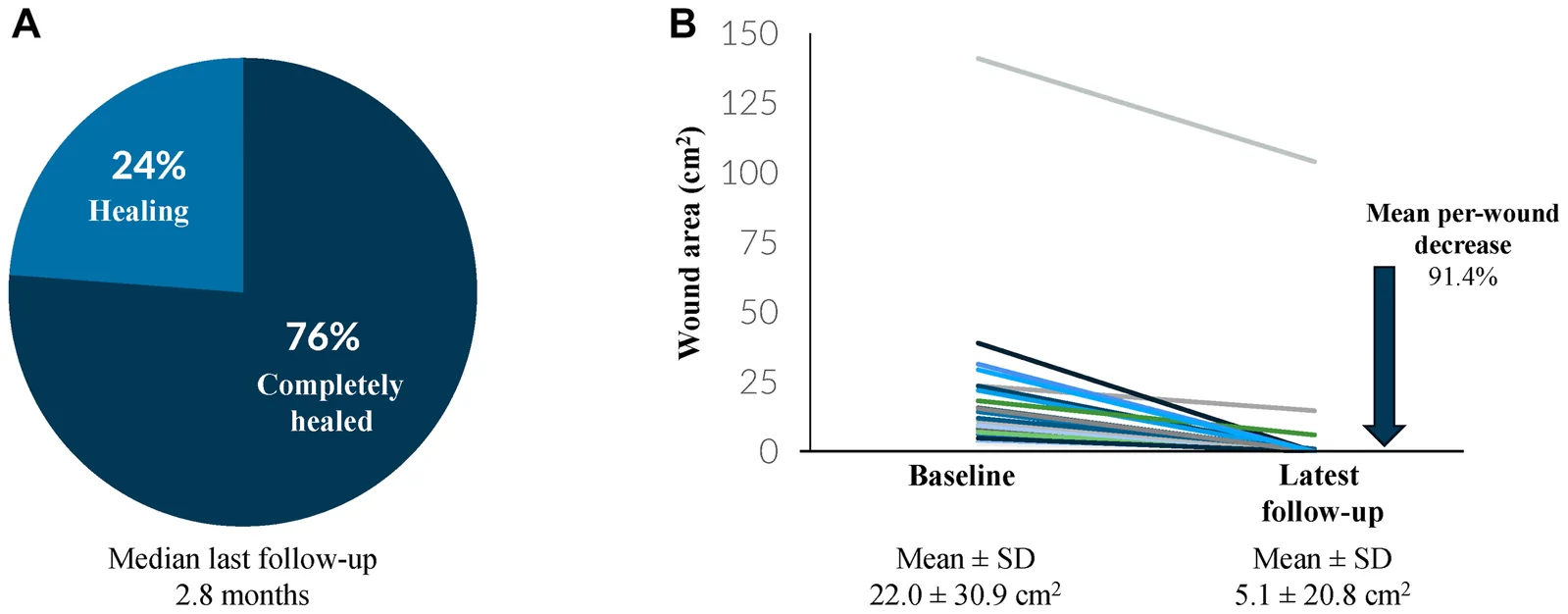
**A,** Proportion of wounds healed or healing at latest follow-up (N = 37). **B,** Wound area at baseline and latest follow-up among wounds with available area measurements (N = 25). *SD*, Standard deviation.

Of the 13 limbs without new stenting and with documented procedural status (2 MT alone, 11 MT plus venoplasty), 11 had documented healing outcomes: six were completely healed—including both limbs treated with MT alone—and five demonstrated partial healing, all meeting the definition of clinical success. Complete healing appeared to occur irrespective of baseline stent status: two of five limbs with a baseline stent and four of eight limbs without were completely healed.

## Discussion

Venous wound disease—the most severe manifestation of PTS—affects approximately 1 to 1.5 million individuals in the United States,^15^ with annual management costs estimated at up to $14.9 billion.^16^ Despite this substantial burden, available treatments remain largely palliative. Compression therapy, the cornerstone of management,^17^ is limited by poor adherence and suboptimal long-term healing rates, with up to 20% of wounds remaining unhealed at 2 years.^18^^,^^19^ Alternative interventions, including ablation, venoplasty, and stenting, have variable efficacy and similarly fail to address the underlying PTO in many patients.^7^ There is an urgent need for treatment modalities that directly target the underlying obstruction and promote durable wound resolution.

This multicenter retrospective analysis adds to a growing body of evidence supporting MT as a means of addressing C6 venous disease secondary to PTO.^11^^,^^12^^,^^14^ Mechanical extirpation of chronic obstructive material—combined with standard wound care and indicated adjunctive endovenous interventions—was associated with high rates of wound healing, with more than 75% of wounds completely resolved and mean per-wound area reduction of 91.4%. The speed of healing is particularly notable: 76% of wounds fully resolved at a median of 2.8 months, compared with published healing rates of 50% to 87% at 6 to 12 months for compression therapy alone in highly selected, compliant populations.^18^, ^19^, ^20^ More than half of the cohort had been referred only after failure of conservative wound care, with a median symptom duration of 13.0 (IQR, 6.3-45.0) months, suggesting that wound care alone was insufficient to drive the observed outcomes.

The data also point to MT itself, rather than adjunctive endovenous interventions, as a meaningful driver of healing. Of 13 limbs treated with MT alone (n = 2) or MT plus adjunctive venoplasty without new stenting (n = 11), 11 had documented healing outcomes: six were fully healed and five demonstrated partial healing. As balloon venoplasty alone has limited durability in chronic venous lesions without stent scaffolding,^7^ the healing observed in this subgroup likely reflects the contribution of MT-mediated thrombus removal. Complete healing in this subgroup was observed irrespective of baseline stent status or adjunctive venoplasty, supporting MT as a meaningful contributor to healing.

Beyond thrombus extraction, MT may also fracture or modify post-thrombotic webs and chronic intraluminal changes, enhancing luminal compliance and improving responsiveness to adjunctive venoplasty. This mechanism is supported by pre- and postprocedural venographic and IVUS imaging demonstrating restoration of luminal patency following thrombus extirpation.^11^^,^^12^ This potential mechanical remodeling effect may partially explain the favorable outcomes observed and supports a broader therapeutic role for MT in complex post-thrombotic disease.

These findings should be interpreted within the context of several important limitations, including the retrospective nature and small sample size of this study. Patency was also not formally assessed as a study endpoint. Follow-up was short and not protocol-driven across centers, precluding assessment of long-term durability and recurrence, and wound care protocols were not unified across sites.

Despite these limitations, the failure of conservative wound care before referral and the favorable outcomes in the 13 limbs treated without new stenting argue against wound care or stenting alone fully accounting for the results observed. The favorable outcomes are particularly noteworthy given the long-standing, refractory nature of disease in this cohort. These findings provide meaningful preliminary evidence supporting MT's role in venous wound management secondary to PTO and form the basis for larger prospective investigation.

## Conclusions

In this multicenter retrospective analysis, all patients with venous wounds secondary to PTO demonstrated wound improvement following MT with or without adjunctive endovenous interventions; over three-quarters of wounds completely healed at a median follow-up of 2.8 months. These findings suggest that MT may play an important role in addressing the underlying venous pathology driving C6 disease. Although further research is needed to confirm these outcomes and refine treatment protocols, this study supports MT as a meaningful addition to the therapeutic arsenal for venous wounds secondary to PTO.

## Author Contributions

Conception and design: NM, SA, AB, AM

Analysis and interpretation: NM, SA

Data collection: NM, SA, AB, AM

Writing the article: NM, SA, AB, AM

Critical revision of the article: NM, SA, AB, AM

Final approval of the article: NM, SA, AB, AM

Statistical analysis: NM

Obtained funding: Not applicable

Overall responsibility: NM

## Funding

None.

## Disclosures

N.J.M. is a speaker and consultant for Stryker, W. L. Gore, Boston Scientific Corporation, and Shockwave Medical. S.A. is a speaker and consultant for Stryker, W. L. Gore, and BD Bard. A.B. and A.M. have no conflicts of interest to disclose.
